# primerForge: a Python program for identifying primer pairs capable of distinguishing groups of genomes from each other

**DOI:** 10.21105/joss.06850

**Published:** 2024-09-16

**Authors:** Joseph S. Wirth, Lee S. Katz, Grant M. Williams, Jessica C. Chen

**Affiliations:** 1 Enteric Diseases Laboratory Branch, Centers for Disease Control and Prevention, Atlanta, GA, United States; 2 Oak Ridge Institute for Science and Education, Oak Ridge, TN, United States

## Abstract

In both molecular epidemiology and microbial ecology, it is useful to be able to categorize specific strains of microorganisms in either an ingroup or an outgroup in a given population, e.g. to distinguish a pathogenic strain of interest from its non-virulent relatives. An “ingroup” refers to a group of microbes that are the primary focus of study or interest. Conversely, an “outgroup” consists of microbes that are closely-related to, but have evolved separately from, the ingroup. While whole genome sequencing and downstream phylogenetic analyses can be employed to do this, these techniques are often slow and can be resource intensive. Additionally, the laboratory would have to sequence the whole genome to use these tools to determine whether or not a new sample is part of the ingroup or outgroup. Alternatively, polymerase chain reaction (PCR) can be used to amplify regions of genetic material that are specific to the strain(s) of interest. PCR is faster, less expensive, and more accessible than whole genome sequencing, so having a PCR-based approach can accelerate the detection of specific strain(s) of microbes and facilitate diagnoses and/or population studies.

## Statement of need

In order to perform PCR, a pair of DNA primers capable of amplifying a region of interest is required. Traditional primer design involves the selection of a target region of DNA to amplify, followed by primer pair selection and subsequent validation of the primer pair. Identifying a good pair of primers and a suitable target region often requires several iterations of the primer design process, which can be tedious and time consuming.

primerForge seeks to assist biologists with the process of primer design. Instead of requiring the identification of specific target sequences (as is required with existing tools), primerForge identifies all suitable pairs of primers capable of producing PCR products of a specific size in a set of whole genome sequences. Optionally, it can also filter those primer pairs and limit the output to primer pairs that can be used to distinguish one set of genomes from another set of genomes via PCR amplification. primerForge relies on the khmer package to extract k-mers from genomic sequences and the primer3-py package to evaluate specific characteristics of primer pairs including melting temperature, hairpin potential, and dimer formation (Crusoe et al., 2015; Untergasser et al., 2012). It also uses *in silico* PCR via the isPcr program to validate and filter the primer pairs (Khun et al., 2012).

There are many use cases for what primerForge offers. One use case would be surveillance of an outbreak clone of a particular pathogen. A laboratory could develop a set of PCR reactions to track the population of this outbreak clone which could help inform if the population were to grow, shrink, or migrate.

## Comparing primerForge to swga2

Another software package, swga2, was developed to choose sets of primers that selectively amplify PCR products in one set of genomes (the ingroup) but not in another set of genomes (the outgroup) (Dwivedi-Yu et al., 2023). Given this similarity, it was important to compare the performance of primerForge to that of swga2. To do this, the sequence files listed in Table 1 were used as inputs. The default parameters were used except that the melting temperature range was set at 55°C to 68°C and only a single processor was used. The primer pairs identified by swga2 and primerForge were evaluated using isPcr with the following additional parameters: -tileSize=8, -minGood=8, and -minPerfect=8. These parameters were necessary because the primers identified by swga2 were too short to use the default value of 15. The results of these comparisons are shown in Table 2.

Although many of the primer pairs predicted by primerForge were not validated by isPcr, this can be attributed to the fact that primerForge allows primer pairs to produce PCR products in the outgroup provided they are outside of the user-specified range. For example, all of the 847,045 primer pairs identified by primerForge in the *E. coli* dataset that were compatible with, but not validated by, isPcr were predicted to produce a PCR product in one or more of the outgroup genomes. Similarly, all 154 primer pairs identified by primerForge but not validated by isPcr in the *M. mycoides* dataset were not validated for the same reason. The decreased number of optimized primer pairs can be attributed to the fact that the isPcr parameters tileSize, minGood, and minPerfect were set to low values in order to directly compare the results of primerForge with those produced by swga2.

In addition to the improvements observed in Table 2, primerForge also allows the user to specify desired PCR product size ranges, specify primer size ranges, specify melting temperature ranges, specify the allowed difference in melting temperature between the forward and reverse primers, exclude certain PCR product sizes from the outgroup genomes, and use sequences in genbank file format. Both primerForge and swga2 can take advantage of parallel processing, which speeds up runtimes. For example, primerForge ran on the *E. coli* dataset in 37 minutes and 7 seconds and used 10.07 GB RAM when using 8 cpus.

## Figures and Tables

**Table 1:** Datasets used to compare primerForge to swga2.

Dataset	Name	NCBI Accession	Group^1^
plasmid^2^	pcDNA	not provided	ingroup
plasmid^2^	pLTR	not provided	outgroup
*M. mycoides* ^3^	*Mycoplasma mycoides* subsp. mycoides str. KH3J	GCF_003034305.1	ingroup
*M. mycoides* ^3^	*Mycoplasma mycoides* subsp. mycoides str. B345/93	GCF_003034275.1	ingroup
*M. mycoides* ^3^	*Mycoplasma mycoides* subsp. mycoides str. Gemu Goffa	GCF_003034345.1	ingroup
*M. mycoides* ^3^	*Mycoplasma mycoides* subsp. capri str. GM12	GCF_900489555.1	outgroup
*M. mycoides* ^3^	*Mycoplasma mycoides* subsp. capri str. 80/93	GCF_018389745.1	outgroup
*E. coli*	*Escherichia coli* O157 str. 644-PT8	GCF_001650295.1	ingroup
*E. coli*	*Escherichia coli* O157 str. AR-0428	GCF_008727175.1	ingroup
*E. coli*	*Escherichia coli* O157 str. FDAARGOS_293	GCF_002208865.2	ingroup
*E. coli*	*Escherichia coli* K12 str. MG1655	GCF_000005845	outgroup
*E. coli*	*Salmonella enterica* subsp. enterica serovar Typhimurium str. LT2	GCF_000006945	outgroup
SARS-CoV-2	SARS-CoV-2 isolate human/USA/MA_MGH_00230/2020	MT520374	ingroup
SARS-CoV-2	SARS-CoV-2 isolate human/USA/MA_MGH_00229/2020	MT520263	ingroup
SARS-CoV-2	SARS-CoV-2 isolate human/USA/MA_MGH_00257/2020	MT520479	ingroup
SARS-CoV-2	SARS-CoV-2 isolate Wuhan-Hu-1	NC_045512	outgroup

1 The group indicates which set of genomes should be distinguished from each other within each dataset. The “ingroup” indicates genomes that should produce PCR products when amplified with the primer pairs identified by the software. The “outgroup” indicates genomes that should that should not produce PCR products, or should produce PCR product sizes outside a user-specified range.

2 The plasmid dataset is provided as an example in the swga2 repository.

3 The *M. mycoides* dataset is provided as an example in the primerForge repository.

**Table 2:** Comparing swga2 vΘ.Θ.1 to primerForge v1.3.5

Program	Dataset	Runtime (mm:ss)	RAM (GB)	Primer Pairs^1^	isPcr-compatible Pairs^2^	Validated Pairs^3^	Optimized Pairs^4^
swga2	plasmid	23:21	0.136	94	22	22	11
primerForge	plasmid	00:10	0.054	3,004	3,004	2,977	2,934
swga2	*M. mycoides*	05:13	0.221	run failed	NA	NA	NA
primerForge	*M. mycoides*	02:25	1.490	1,096	1,096	942	884
swga2	*E. coli*	21:10	4.452	run failed	NA	NA	NA
primerForge	*E. coli*	81:09	10.645	1,150,858	1,148,425	301,380	125,357
swga2	SARS-CoV-2	10:38	0.141	63	7	0	0
primerForge	SARS-CoV-2	00:20	0.118	15	15	15	15

1 The number of primer pairs identified by the program.

2 The number of primer pairs that generated PCR products with isPcr.

3 The number of primer pairs that produced a PCR product in every ingroup genome and no products in any of the outgroup genomes.

4 The number of valid primer pairs that produced exactly one PCR product in each ingroup genome.
